# Effect of Different Classes of Antihypertensive Drugs on Endothelial Function and Inflammation

**DOI:** 10.3390/ijms20143458

**Published:** 2019-07-14

**Authors:** Isabella Viana Gomes Silva, Roberta Carvalho de Figueiredo, Danyelle Romana Alves Rios

**Affiliations:** Campus Centro Oeste Dona Lindu—Universidade Federal de São João del-Rei, Chanadour 400, Brazil

**Keywords:** hypertension, endothelial dysfunction, beta-blockers, calcium channel blockers, inhibitors of angiotensin converting enzyme and angiotensin receptor blockers

## Abstract

Hypertension is characterized by structural and functional changes in blood vessels that travel with increased arterial stiffness, vascular inflammation, and endothelial dysfunction. Some antihypertensive drugs have been shown to improve endothelial function and reduce levels of inflammatory markers regardless of the effect of blood pressure lowering. Third-generation β-blockers, such as nebivolol and carvedilol, because they have additional properties, have been shown to improve endothelial function in patients with hypertension. Calcium channel antagonists, because they have antioxidant effects, may improve endothelial function and vascular inflammation.The Angiotensin Receptor Blocker (ARBs) are able to improve endothelial dysfunction and vascular inflammation in patients with hypertension and other cardiovascular diseases. Angiotensin converting enzyme (ACE) inhibitors have shown beneficial effects on endothelial function in patients with hypertension and other cardiovascular diseases, however there are few studies evaluating the effect of treatment with this class on the reduction of C-reactive protein (CRP) levels. Further studies are needed to assess whether treatment of endothelial dysfunction and vascular inflammation may improve the prognosis of patients with essential hypertension.

## 1. Introduction

Endothelium plays an essential role in vascular function through several mechanisms, including the synthesis and release of substances that act in an autocrine and/or paracrine form. Endothelial dysfunction may be characterized by changes in the production or bioavailability of vasoactive molecules, especially vasodilating molecules such as nitric oxide (NO), and vasoconstrictors such as angiotensins and endothelin. The effects of these and other molecular changes in vascular reactivity lead to increased oxidative stress and activation of proinflammatory signaling pathways that cyclically contribute to the process of endothelial dysfunction [1,2].

The hypertension induces structural and functional changes in blood vessels that increase arterial stiffness, vascular inflammation, and endothelial dysfunction, similarly, endothelial dysfunction and vascular inflammation may also contribute to increased blood pressure [1,3,4]. During the inflammatory process, the organism produces molecules, such as C-reactive protein (CRP) and free radicals, which are able to decrease the production or bioavailability of NO leading to endothelial dysfunction [1]. Inflammatory markers such as CRP, as well as endothelial dysfunction, are associated with cardiovascular risk factors and may predict cardiovascular events such as acute myocardial infarction and encephalic vascular accident [2,5,6,7,8,9]. In this sense, the improvement of endothelial function and vascular inflammation may represent an important target for treatment with antihypertensive drugs in patients with hypertension.

In this article, we provide an overview about the effect of treatment with different classes of antihypertensive drugs on endothelial function and vascular inflammation.

## 2. Hypertension and Antihypertensive Drugs

Hypertension is a chronic multifactorial condition characterized by a sustained increase in systolic and diastolic blood pressure (BP) levels [3,10]. There is a continuing association between increased blood pressure and increased risk of cardiovascular disease, however, for public health decision making, BP can be categorized. According to the “Guideline for the Prevention, Detection, Evaluation and Management of Blood Hypertension in Adults (2017)” the mean BP can be categorized into four levels: normal, elevated, and stage 1 or 2 hypertension [3].

Hypertension represents the most important modifiable risk factor for stroke, heart failure, and chronic kidney disease, contributing significantly to the global burden of cardiovascular disease [1,10]. It is often associated with metabolic disorders, functional and/or structural alterations of target organs, and is aggravated by the presence of other risk factors [1,11]. In Brazil, hypertension affects approximately 30% of adult individuals [12]. Baseline results from the longitudinal study of adult health, 2008–2010, (ELSA-Brasil) showed that the prevalence of hypertension was approximately 37%. In addition, the prevalence of hypertension was higher among men (42%) than in women (34%) [13].

The initial assessment of a patient with hypertension is important to assist in choosing an effective therapy, all patient risk factors should be taken into account, which includes identification of damage to target organs as well as possible secondary causes of hypertension. The treatment of hypertension should focus on the overall health of the patient, focusing on reducing the risk of future cardiovascular events. As BP and the risk of cardiovascular disease increase, care with hypertension treatment should be intensified [3].

Antihypertensive drugs, as well as lifestyle modification, provide the primary basis for the treatment of high BP. Several classes of medications are available for the treatment of hypertension. Medications that have been shown to have a better effect in reducing the risk of developing cardiovascular disease should be used preferentially. Therefore, the main drugs used in the treatment of hypertension include thiazide diuretics, inhibitors of angiotensin converting enzyme (ACEI) and angiotensin receptor blockers (ARBs), and calcium channel blocker (CCB). Evidence is controversial to support the initial use of beta-blockers for hypertension in the absence of specific cardiovascular comorbidities [3].

Several strategies can be used for the initial pharmacological treatment of hypertension. Many patients start treatment with only one medication, however in patients with stage 2 hypertension, one should consider starting with two medications of different classes. When single-drug pharmacological treatment is appropriate, patient comorbidities (e.g., diabetes mellitus, heart failure, acute myocardial infarction, and chronic kidney disease) should be considered for which specific classes of anti- hypertensive drugs to reduce BP. In addition, other patient-specific factors, such as age, use of multiple medications, adherence to treatment, drug interactions, and costs, should be considered. Many patients who start treatment with only one drug may subsequently need two or more drugs of different pharmacological classes for BP reduction. Knowledge of the mechanism of action of each antihypertensive drug is important. Therapeutic regimens with two or more drugs with complementary activity in which a second antihypertensive drug is used to block compensatory responses to the starting medicament or to affect different hypertensive mechanisms may result in the additive reduction of BP [3].

Diuretics increase urinary flow and sodium excretion and are used to adjust the volume and/or composition of body fluids [10]. They are divided into groups that have different mechanisms of action and pharmacological characteristics: carbonic anhydrase inhibitors, osmotic diuretics, loop diuretics, thiazide diuretics, potassium-sparing diuretics, and mineralocorticoid receptor antagonists [10]. Thiazide diuretics have been used for the treatment of hypertension since the late 1950s and continue to be one of the most important groups of drugs used to reduce blood pressure due to its effectiveness and cost-effectiveness [14]. The “antihypertensive and lipid-lowering treatment to prevent heart attack trial” (ALLHAT) compared the use of different classes of antihypertensive drugs, chlortalidone, amlodipine, and lisinopril, in 33,357 patients with hypertension. The overall results of the study showed that there was no difference between reducing the risk of coronary artery disease among patients taking amlodipine and lisinopril compared to treatment with chlorthalidone and that total mortality was similar in all three groups. However, therapy using diuretics was superior to CCB and an ACEI in preventing one or more major forms of cardiovascular disease, including heart failure and, in some cases, stroke [15].

The pharmacological properties of beta-blockers can be explained in large part from the responses produced by the receptors in various tissues and the activity of the sympathetic nerves that innervate these tissues. Thus, beta-receptor blockade has relatively little effect on the normal heart of a resting individual, but exerts significant effects when sympathetic control of the heart predominates, such as during exercise or stress [10,16,17,18]. The main therapeutic effects of beta-blockers occur in the cardiovascular system. It is important to distinguish these effects in normal individuals from those in individuals with cardiovascular diseases, such as hypertension. Because catecholamines exert positive chronotropic and inotropic actions, beta-receptor antagonists decrease heart rate and myocardial contractility [10,16,17,18]. When tonic stimulation of beta receptors is low, this effect is modest. However, when the sympathetic nervous system is activated, the beta-blockers attenuate the expected increase in heart rate. Short-term administration of a non-selective beta-blocker decreases cardiac output, peripheral resistance increases to maintain blood pressure, as a consequence of vascular beta-2 receptor blockade and compensatory reflexes, as increased sympathetic nervous system activity, resulting in activation of the alpha arteriolar receptors [16,17,18]. However, with prolonged use of beta-blockers, total peripheral resistance returns to baseline or decreases in patients with hypertension. In general, beta blockers do not reduce blood pressure in patients with normal blood pressure, however, this effect is observed in patients with hypertension. Despite its wide use, the mechanisms responsible for this important clinical effect are not well elucidated. The release of renin from the juxtaglomerular apparatus is also stimulated by the sympathetic nervous system through β1 receptors, and this effect is reduced by beta blockers. However, the relationship between this phenomenon and the drop in blood pressure is not well understood [10,16,17,18]. Beta-blockers can be classified into three groups: first-generation non-selective beta-blockers, second-generation beta-selective beta-blockers, and beta-blockers with additional third-generation cardiovascular actions [10]. Third-generation drugs, in addition to previous actions, have vasodilatory effects by different mechanisms: concomitant alpha-1 adrenergic receptor blockade and increased synthesis and release of nitric oxide in the vascular endothelium [10,19]. Beta-blockers are indicated for the treatment of hypertension, particularly in patients with specific indications such as high risk of coronary heart disease and heart failure, or in patients with acute myocardial infarction, patients with stable angina, diastolic and systolic heart failure, hyperadrenergic (increased sympathetic activity) and supraventricular arrhythmias. Under these particular conditions, the effects of β-blockers may provide benefits in addition to reducing blood pressure [16].

The CCB reduces blood pressure by relaxing arteriolar smooth muscle and decreasing peripheral vascular resistance [20]. Voltage sensitive L-type calcium channels control the entry of extracellular calcium into the smooth and cardiac muscle cells and into the sinoatrial node and atrioventricular node cells in response to electrical depolarization [20]. Increased concentrations of calcium in the cytoplasm causes increased contraction of vascular smooth muscle and cardiac muscle cells. Calcium channel blockers exert their effects through their binding to the α1 subunit of the L-type calcium channels, reducing calcium flow through the channel [10]. The CCB is indicated as an alternative to initial therapy when thiazide diuretics are not tolerated or in combination with Angiotensin converting enzyme ACE inhibitors and ARBs as adjunctive therapy [3].

ACE inhibitors and ARBs act in the renin angiotensin system (RAS), inhibiting the production and action of angiotensin II (Ang II), respectively. RAS is a complex system composed of peptides, enzymes and receptors, responsible for broad functions in the body, among them the control of BP [21,22]. In this system, renin, an enzyme released by the juxtaglomerular cells of the kidneys, acts on the angiotensinogen produced by the liver, converting it into angiotensin I (Ang I), which is immediately transformed by the action of the angiotensin converting enzyme (ACE) in Ang II [21]. Ang II, one of the main effectors of RAS, acts via the angiotensin receptor type 1 (AT1) and angiotensin receptor type 2 (AT2). On binding to the AT 1 receptor it has vasoconstriction, sodium and water retention, aldosterone release, and proliferative and inflammatory effects in various tissues [23]. In contrast, the binding of Ang II to AT 2 receptors exerts generally opposite effects to those mediated by AT 1 receptors [21]. In adults with hypertension and chronic kidney disease, treatment with an ACE inhibitor or ARB when ACE inhibitors are not well tolerated is indicated to delay the progression of renal disease. In some patients with hypertension and diabetes mellitus, ACE inhibitors and ARBs are also indicated to prevent renal damage [3].

## 3. Endothelial Dysfunction, Hypertension, and Inflammation

The vascular system is composed of arteries, arterioles, capillaries, venules, and veins. All blood vessels, except capillaries, are formed by three layers that undergo modifications depending on their function. The outermost layer is the adventitia, consisting mainly of connective tissue, the middle layer is formed mainly by smooth muscle cells and components of elastic tissue, and the inner layer is the intima consisted of the endothelium and subendothelium [10,24]. Each of these layers exhibits specific histological, biochemical, and functional characteristics and, therefore, each contributes uniquely to maintain vascular homeostasis and to regulate vascular response to stress or injury, in that sense, the proportion of these layers in each vessel is directly related to the function that this vessel executes [24].

The endothelium consists of a monolayer of cells, called endothelial cells, that coat the entire cardiovascular system and play an essential role in vascular function through the synthesis and release of substances that act in an autocrine and/or paracrine manner [25,26]. Initially, it was considered only as a passive barrier between blood and tissues, however, it has been shown that this tissue performs ample important functions in the body [27]. The endothelium regulates vascular tone by balancing the production of vasodilatory molecules, such as nitric oxide (NO), and vasoconstrictor, such as endothelin 1 (ET-1) and Ang II, controls blood flow through the production of regulating factors platelet activity, the coagulation cascade, and the fibrinolytic system and, in addition, regulates the production of cytokines and adhesion molecules that modulate and direct the inflammatory process [28]. In addition, recent studies have shown that endothelial cells are capable of producing catecholamines that may contribute to the process of endothelial dysfunction [29]. Other studies suggest a reciprocal relationship between endothelial function and the activity of the sympathetic nervous system. An acute increase in sympathetic activity has been shown to cause a decrease in endothelial function [30,31].

MicroRNAs (miRs) are small, generally non-coding RNAs that regulate gene expression via post-transcriptional degradation or translational repression. The miRs are fundamental regulators of numerous biological processes [32]. The fundamental importance of miRs in endothelial physiology is clearly indicated by the phenotype obtained after EC specific inactivation of Dicer, an enzyme involved in biogenesis and miR processing that cleaves miRs precursors to mature forms [33]. The lack of Dicer in the endothelium leads to altered expression of fundamental regulators of endothelial function, including endothelial nitric oxide synthase (eNOS), vascular endothelial growth factor (VEGF) 2 receptor, and interleukin-8 [32].

The term endothelial dysfunction is typically used to refer to abnormalities in the production or bioavailability of endothelium-derived nitric oxide and the resulting deleterious changes in vascular reactivity, encompassing both the irregular production of messenger molecules and the expression of pro-inflammatory adhesion molecules [34]. Endothelial dysfunction is characterized by a pro-inflammatory and prothrombotic endothelium with compromised vasodilator responses [1,26] and represents a central component of vascular changes that occur in the development of hypertension [1,25,35,36].

Hypertension causes vascular injury through different factors, which affect prematurely the small vessels and, later, the great vessels [11]. The relationship between pressure levels and vascular changes is based on a cyclical and evolutionary process in which some disturbance in the cardiovascular system increases the neurohumoral activity with pressure elevation and this, in turn, leads to structural and functional changes that, as a consequence, promotes greater vascular resistance and, therefore, higher blood pressure.

Several human studies have linked increased vascular inflammation with decreased NO bioavailability, showing that chronic inflammation is related to endothelial dysfunction [3,37,38,39].

The inflammatory process leads to an increase in the number of cells (neutrophils, monocytes), cytokines, and proinflammatory proteins, such as interleukin-6 (IL-6), tumor necrosis factor-α (TNF-α) and CRP. The increase of neutrophils and macrophages in response to inflammation causes a greater synthesis of IL-6 which, in turn, increases the production of CRP in the liver [40,41]. PCR works directly in the NO uptake by decreasing the activity of endothelial nitric oxide synthase (eNOS) in endothelial cells, thereby decreasing NO bioavailability and also increasing the concentration of ET-1 (vasoconstrictor) [41,42]. This decrease in vasodilation causes shear stress and, consequently, greater damage to the blood vessels, thus favoring the process of endothelial dysfunction [41,42]. In addition, CRP stimulates the expression of inflammatory molecules such as ICAM-1, VCAM 1, E-selectin, Monocyte Chemoattractant Protein-1 (MCP-1), and activates cytokine-expressing macrophages and tissue factors that contribute to endothelial injury. Because it has the ability to alter the endothelial cell phenotype and thus contribute to the formation of endothelial lesions, in addition to acting as an inflammatory biomarker, this protein has also been considered a marker of endothelial dysfunction [37,38].

Markers of vascular inflammation and endothelial dysfunction were consistently associated with increased risk of developing cardiovascular disease [5,6]. In a case-control study, Ridker et al. [5] evaluated the risk of cardiovascular events associated with baseline inflammatory markers in 28,263 apparently healthy postmenopausal women. The inflammatory markers were CRPhs, amyloid A, IL-6 and soluble intercellular adhesion molecule type 1 (slCAM-1). Cardiovascular events were defined as death from coronary heart disease, nonfatal myocardial infarction, stroke, or need for coronary revascularization procedures. CRPh has been shown to be a strong and significant predictor of the risk of future cardiovascular events after a mean follow-up period of three years. Pai et al. [6] evaluated plasma levels of soluble TNF Receptors (sTNF-R1 and sTNF-R2), IL-6, and CRP as risk markers for coronary heart disease and acute myocardial infarction among women attending the nurses’ health study (NHS) and men participating in the “Health Professionals Follow-up Study” (HPFS) in a case-control study during 8 years of follow-up. High levels of IL-6 and CRP were significantly associated with an increased risk of coronary disease in both sexes, while high levels of soluble TNF receptors were significantly associated only among women. After adjustments for lipid and non-lipid factors all associations were attenuated, only CRP levels remained significant. They concluded that elevated levels of inflammatory markers, particularly CRP, indicate an increased risk of coronary disease. Ghiadoni et al. [35] evaluated carotid artery mid-intima thickening by ultrasonography, forebrain vascular response by plethysmography after infusion of intrabrachial acetylcholine in 44 patients with essential hypertension diagnosed less than 12 months and who were untreated. They observed that carotid wall thickening was associated with reduced endothelium-dependent vasodilation and suggested that endothelial dysfunction may be involved in early structural arterial changes. Suwaidi et al. [8] showed that in a longitudinal study conducted with 157 patients with coronary artery disease evaluated by the epicardial coronary vasoropathy method, only those with severe endothelial dysfunction suffered cardiovascular events during approximately two years of follow-up. Similarly, Schächinger et al. [9], showed that in 147 patients with hypertension accompanied by a follow-up period of approximately 8 years, evaluated by the epicardial coronary vasoractivity method, there was a significant association between coronary endothelial dysfunction and risk of cardiovascular events. In a prospective multicenter observational study, Maruhashi et al. [39] evaluated flow-mediated vasodilatation (FMV) and brachial pulse wave velocity (PWV) in 462 participants with coronary artery disease during an average follow-up period of 49.2 months. They observed that both FMV and PWV were predictors of cardiovascular events.

These studies support the concept that endothelial dysfunction and vascular inflammation may play an important role in increasing the risk of developing cardiovascular events. In this context, the need to treat these vascular changes that are associated with an increase in blood pressure is increasingly evident [1]. Antihypertensive drugs that, in addition to reducing BP, have additional effects enhancing endothelial function and vascular inflammation could be more effective in reducing cardiovascular risk than antihypertensive drugs that reduce blood pressure only [1,25,43].

## 4. Antihypertensive Drugs, Endothelial Dysfunction, and Inflammation

### 4.1. Inhibitors of Angiotensin Converting Enzyme (ACEI) and Angiotensin Receptor Blockers (ARBs)

The renin angiotensin system (RAS), mainly angiotensin II (Ang II), plays a central role in the decrease of NO production and bioavailability, stimulating the production of free radicals and inflammatory molecules [10,11,12].

The ACE inhibitors and ARBs, by reducing the oxidative and inflammatory effects induced by angiotensin II, may add additional benefits by limiting endothelial dysfunction and vascular inflammation [1,22,25,44]. In addition, ACEI inhibit the degradation of bradykinin (which induces NO release) which results in improved endothelium-dependent vasodilation [1,25,43] and ARBs, by blocking AT1 receptors, favor the binding of Ang II to free AT2 receptors and consequently stimulates synthesis and NO release induced by that receptor [1,45,46].

Randomized clinical trials have shown that treatment with different ACEI may improve endothelial function in patients at high risk of cardiovascular events. The trial on reversing endothelial dysfunction (TREND) study showed that, in comparison to placebo, the six-month treatment with quinapril improved endothelial dysfunction assessed by coronary artery diameter response to intracoronary acetylcholine infusion in normotensive patients with coronary artery disease [47]. In the BANFF trial, the effect of the eight-week treatment with ACE inhibitors (quinapril and enalapril), BRA (losartan) and CBC (amlodipine) on DMF in 80 patients with coronary disease was compared. Only quinapril improved flow mediated dilatation (FMD) compared to baseline. No change was observed with enalapril, losartan, or amlodipine [48].

The beneficial effects of ACEI on endothelial function and vascular inflammation have been demonstrated in patients with essential hypertension. Treatment with cilazapril for two years [49] and lisinopril for three years [33] improved the response to acetylcholine in the subcutaneous microcirculation compared to atenolol [33,49]. Ghiadoni et al. [50] evaluated the effect of ramipril treatment for three months on radial artery FMD before and after intra-arterial infusion of N (G) -monomethyl-L-arginine (L-NMMA). Treatment with ramipril increased radial artery FMD compared to baseline. Galezewska et al. [51] showed that the 12-week treatment with ramipril reduced CRP levels compared to treatment with nebivolol.

Angiotensin II receptor blockers have also been shown to have beneficial effects on endothelial function and vascular inflammation in patients with hypertension. Ghiadoni et al. [52] showed that treatment with candesartan for 12 months improves the release of nitric oxide and reduces endothelin-1 (ET-1) mediated vasoconstriction compared to baseline, as assessed by altered forearm blood flow induced by the intrabrachial infusion of L-NMMA, norepinephrine, ET A/B receptor antagonist TAK 044, sodium nitroprusside, and acetylcholine. Blocking positive Ang II feedback in ET-1 synthesis may explain this beneficial effect of candesartan on the biological activity of ET-1. This effect could prevent or reverse functional and structural cardiovascular alterations attributable to ET-1 [53]. Yasunari et al. [54] showed that the use of ARB reduced CRP levels when compared to the use of calcium channel blockers after eight months of follow-up. Taguchi et al. [55] showed that irbesartan reduced CRP levels and free radicals after four weeks of treatment compared to baseline.

ARBs demonstrated direct effects on inflammatory markers regardless of blood pressure (BP) reduction. The Valsartan-managing blood pressure aggressively and evaluating reductions in hsCRP (Val-MARC) is a prospective study comparing the efficacy of valsartan monotherapy and valsartan associated with hydrochlorothiazide in 1668 hypertensive patients to assess the effect of these two therapeutic regimens on plasma CRP levels and to determine whether or not these effects were dependent on BP reduction. Valsartan monotherapy significantly reduced CRP levels, although the combination of valsartan plus hydrochlorothiazide was more effective in reducing BP. The therapeutic regimen of valsartan and hydrochlorothiazide appears to have neutralized the effects observed on CRP levels obtained when valsaratan was used as monotherapy [56].

The beneficial effect of ACEI and ARBs on endothelial function has also been documented in the renal circulation. Schmieder et al. [57] showed that in patients with hypertension and type 2 diabetes mellitus, treatment with ramipril or telmisaran for nine weeks significantly increased NO activity in the renal endothelium.

They are scarce observational studies that have evaluated the effect of different classes of antihypertensive drugs on endothelial function and vascular inflammation. In a cross-sectional study conducted with baseline participants (2000–2002) of the multi-ethnic study of Atherosclerosis (MESA), among monotherapy participants, CRP levels were lower in those using β-blocker, ACEI, or ARB compared to diuretic users. Among the participants in polytherapy, those who used at least one β-blocker had significantly lower CRP levels compared to participants who did not use this class [58]. Higashi et al. [59] compared the effect of different antihypertensive drugs (calcium channel blocker, ACEI, β-blockers, and diuretics) on the endothelial function of 296 patients with essential hypertension by assessing forearm blood flow after reactive hyperemia and sublingual administration of nitroglycerin. Only ACEIs increased the vascular response of the forearm to reactive hyperemia by increasing NO production in these patients with essential hypertension. Buda et al. [60] demonstrated in a cross-sectional study that treatment with candesartan was associated with lower plasma levels of pentraxin-3 (PTX3) and CRP compared to other classes of antihypertensive drugs (β-blockers, calcium channel blocker, and diuretics) in 365 patients with essential hypertension. On the other hand, Vidal et al. [61] showed in a prospective population-based study conducted in Switzerland that the use of ARB was not associated with reduced levels of inflammatory markers when compared with non-users (users of other classes of antihypertensive drugs).

In conclusion, randomized clinical trials have shown that treatment with BRA is able to improve endothelial dysfunction and vascular inflammation in patients with hypertension and other cardiovascular diseases compared to placebo and compared to treatment with other classes of antihypertensive drugs. Regarding ACEIs, clinical trials have shown beneficial effects on endothelial function in patients with hypertension and other cardiovascular diseases, however, there are few studies evaluating the effect of treatment with this class on the reduction of CRP levels (Table 1). There are few observational studies that have evaluated the effect of different classes of antihypertensive drugs on the levels of inflammatory markers and endothelial dysfunction and the results are not yet consensual.

### 4.2. Calcium Channel Blocker (CCB)

The CCB may also have pleiotropic effects leading to an improvement in endothelial function [44]. Endothelial cells do not express voltage-dependent calcium channels, so improvements in endothelial function observed with the use of this class are unlikely to be calcium dependent [25,62]. Instead, these drugs appear to have antioxidant effects that can protect endothelial cells from free radicals, thereby improving the bioavailability of NO and consequently endothelial function [25,63]. The antioxidant activity of BCCs is attributed to their high lipophilicity and to a chemical structure that facilitates electron donation mechanisms and resonance stabilization that inhibits free radicals [64,65]. Some CCBs have also been shown to modify endothelial function, increasing endothelial nitric oxide synthase (eNOS) activity, resulting in increased NO production [64,65,66].

The evaluation of nifedipine and cerivastatin sodium on recovery of endothelial function I (ENCORE I) evaluated the effect of six months of treatment with nifedipine, cerivastatin, the combination of nifedipine and cerivastatin, and placebo treatment through changes in coronary diameter measured by quantitative angiography after coronary infusion of acetylcholine in patients with coronary disease [67]. The results showed that only treatment with nifedipine resulted in an improvement in the response to acetylcholine compared to the placebo group. Such beneficial effects were confirmed in the ENCORE II study which demonstrated that treatment with nifedipine for two years improved endothelial function in patients with coronary disease compared to placebo [68].

The beneficial effects of BCC on endothelial function have also been observed in patients with hypertension. Nifedipine has been shown to reduce markers of oxidative stress and to improve the bioavailability of NO in patients with essential hypertension, an effect probably determined by its antioxidant activity [25,69]. Similarly, in patients with essential hypertension, treatment with nifedipine for one year improved the structure and function of the small subcutaneous gluteal arteries assessed by gluteal subcutaneous biopsy compared to treatment with atenolol [70]. Sudano et al. [71] showed that after 24 weeks of treatment, nifedipine reduced ET-1-induced vasoconstriction and improved endothelium-dependent vasodilation compared to baseline in patients with essential hypertension. Celik et al. [72] have shown that in patients with newly diagnosed essential hypertension, amlodipine or valsartan have been shown to decrease levels of inflammatory markers (CRP) and endothelial function (Endocan) compared to baseline. Kim et al. [73] showed that patients with type 2 diabetes and hypertension treated with amlodipine or valsartan had reduced levels of oxidative stress markers compared to baseline. In patients with essential hypertension, the four-week treatment with lecardipine increased the number of endothelial progenitor cells and reduced levels of interleukin (IL)-18, Monocyte chemoattractant protein 1 (MCP-1), and CRP compared to placebo [74].

In summary, evidence from the literature shows that calcium channel antagonists, especially nifedipine, improve endothelial dysfunction, however, few studies have evaluated the effect of this class on inflammatory markers levels. Calcium channel blockers, because they have antioxidant properties, can attenuate endothelial dysfunction by restoring NO availability (Table 2).

### 4.3. Beta (β) Blockers

There are few studies that have been designed to evaluate the effect of treatment with β-blockers on endothelial dysfunction or inflammation [25,53]. First- or second-generation β-blockers do not appear to improve arterial stiffness, inflammatory cytokine expression, NO-dependent vasodilation, or oxidative stress in patients with hypertension [21,40,73]. Treatment with atenolol for one to three years did not improve endothelium-dependent vasodilator responses to acetylcholine or bradykinin compared to baseline [69]. Schiffrin et al. [70] showed that treatment with atenolol for one year did not improve the structure and function of small subcutaneous gluteal arteries.

Some third-generation β-blockers, because they have additional properties, have been shown to have beneficial effects on endothelium in patients with hypertension [53]. Nebivolol, a selective beta-β-blocker, has vasodilatory properties through the activation of the L-Arginine-NO pathway and can improve endothelial function in patients with hypertension [25,45,75]. Intra-arterial infusion of high concentrations of nebivolol in the forearm microcirculation of healthy volunteers caused vasodilation, an effect inhibited by L-NMMA, indicating that, in acute treatment, nebivolol causes NO-dependent vasodilation [75]. In addition, in an experimental study, Mason et al. [76] demonstrated that nebivolol inhibits the activity of NAD (P) H oxidase, showing that this drug improves endothelial dysfunction through an antioxidant mechanism independent of β1 receptor blocking activity. This effect may reduce a major source of oxidative stress in hypertension, but needs further clarification in human studies [53]. Carvedilol, a nonselective β-blocker with additional α 1 -adrenergic receptor antagonist activity, has also been shown to exert important beneficial actions in endothelial dysfunction through an antioxidant effect [10,25]. Carvedilol was able to improve endothelial function as assessed by FMD in a group of patients with hypertension and diabetes mellitus compared to placebo [77,78]. More studies are needed to clarify the molecular mechanisms by which carvedilol exerts its antioxidant activity, leading to improved endothelial dysfunction [53].

In summary, the first and second generation β-blockers have not demonstrated beneficial effects on endothelial dysfunction and inflammation. However, some third-generation β-blockers that have additional pharmacological properties have shown benefits in reducing endothelial dysfunction in patients with hypertension. There are few studies evaluating the effects of these drugs on the levels of inflammatory markers (Table 3).

### 4.4. Diuretics

No studies were found that evaluated the effect of treatment with diuretics on endothelium-dependent vasodilation. Some studies comparing the significance of classes of antihypertensive drugs on reference rates are not successful after the use of diuretics in relation to the use of other classes of antihypertensives [58,60]. In addition to having no beneficial effects on inflammatory markers levels, Eriksson et al. [79] showed that 12-week treatment with diuretics was associated with higher levels of CRP over treatment with candesartan or placebo. Ridker et al. [56] showed in Val-MARC that treatment with valsartan and hydrochlorothiazide neutralized the beneficial effects of treatment alone with valsartan in endothelial function in patients with stage 2 arterial hypertension (Table 4).

## 5. Conclusions

Endothelial dysfunction and vascular inflammation are considered markers of early risk of atherosclerosis and are associated with an increase in the incidence of cardiovascular events [5,6,9]. It involves the reduction of NO availability, together with the release of vasoconstrictor molecules such as ET-1 and Ang II, increased production of pro-inflammatory molecules, and production of free radicals [1,2].

Studies have shown that endothelial dysfunction and vascular inflammation can be attenuated with the use of some specific classes of antihypertensive drugs. ACEI and ARBs have shown better endothelial function and reduced levels of inflammatory markers, and these effects are probably due to the increased bioavailability of NO and the prevention of oxidative stress and vascular inflammation induced by Ang II. CCB can restore endothelium-dependent vasodilation in patients with hypertension, improving NO bioavailability through antioxidant activity. First- and second-generation β-blockers have not shown beneficial effects on endothelial function and vascular inflammation, however, some third-generation β-blockers have additional properties that may generate beneficial effects on endothelial function in patients with hypertension (Table 5).

Although there is consistent evidence from randomized clinical trials showing that endothelial function and vascular inflammation can be improved by appropriate antihypertensive treatment, observational studies evaluating this association are still scarce and conflicting. Further studies are needed to assess whether these benefits observed with specific use of some classes of antihypertensive drugs also apply to real-world studies. Moreover, further studies are needed to assess whether these effects independently provide a better prognosis for patients with arterial hypertension and whether they may in fact add greater benefits to clinical practice.

## Figures and Tables

**Table 1:** Randomized clinical trials showing the effect of treatment with angiotensin converting enzyme inhibitors (ACEI) and angiotensin II receptor blockers (ARBs) on serum C-reactive protein levels and endothelial function.

Author	Drugs	Population	Duration	Results
Galezewska et al.	Ramipril × nebivolol	Hypertension	12 weeks	Ramipril reduced CRP levels compared to nebivolol.
Ridker et al. (Val-MARC)	Hydrochlorothiazide + Valsartan vs. Valsartan	Hypertension	6 weeks	Treatment alone with valsartan reduced CRP levels.
Matsuda et al.	ARB × CCB	Hypertension	3 years	ARB reduced CRP levels compared to CCB.
Ghiadoni et al.	Lisopril	Ventricular hypertrophy	3 years	Lisopril improved endothelial function compared to baseline.
Elyas et al.	Cilazapril × Atenolol	Hypertension	2 years	Cilazapril improved endothelial function compared to atenolol treatment.
Rizzoni et al.	Ramipril	Hypertension	3 months	Ramipril improved endothelial function compared to baseline.
Ghiadoni et al.	Candesartan	Hypertension	12 months	Candesartan improved endothelial function compared to baseline.
Schiffrin et al.	Losartan	Hypertension	1 years	Losartan improved endothelial function compared to baseline.
Schmieder et al.	Telmisaran	Hypertension and DM	9 weeks	Telmisaran improved endothelial function compared to baseline.

CRP: C Reactive Protein; ARB: Angiotensin II receptor blockers; CCB: Calcium channel blockers; DM: Diabetes *mellitus* 2.

**Table 2:** Randomized clinical trials showing the effect of treatment with calcium channel blockers on serum C-reactive protein levels and endothelial function.

Author	Drugs	Population	Duration	Results
Celik et al.	Anlodipine	Hypertension	4 weeks	Amlodipine reduced CRP levels compared to baseline.
De Ciuceis et al.	Lecardipine × placebo	Hpertension	4 weeks	Lecardipine reduced CRP levels compared to placebo.
ENCORE I	Nifedipine × placebo	Coronary disease	6 months	Nifedipine improved the inflatable function compared to placebo.
Lüscher et al.	Nifedipine × placebo	Coronary disease	2 years	Nifedipine improved the inflatable function compared to placebo.
Schiffrin et al.	Nifedipine × atenolol × placebo	Hypertension	1 year	Nifedipine improved the endetelial function compared to atenolol and placebo.
Sudano et al.	Nifedipine	Hypertension	24 weeks	Nifedipine improved esophelial function compared to baseline.

CRP: C-reactive protein; ARB: Angiotensin II receptor blockers; CCB: Calcium channel blockers.

**Table 3:** Studies showing the effect of beta-blocker treatment on serum C-reactive protein levels and endothelial function.

Author	Drug	Population	Duration	Results
Jekell et al.	Atenolol × ibesartan	Hypertension	48 weeks	Atenolol did not reduce CRP levels
Ghiadoni et al.	Atenolol	Hypertension	3 year	Atenolol did not improve endothelial dysfunction compared to baseline.
Bank et al.	Carvedilol × metropolol	Hypertension	1 year	Carvedilol improves endothelial function compared to metoprolol.
Cockcroft et al.	Nebivolol	Healthy volunteers	-	Acute treatment nebivolol causes NO-dependent vasodilation
Mason et al.	Nebivolol	Healthy volunteers	-	Nebivolol inhibits the activity of NAD (P) H oxidase

CRP: C-reactive protein.

**Table 4:** Randomized clinical trials showing the effect of treatment with diuretics on serum levels of C-reactive protein.

Author	Drugs	Population	Duration	Results
Rahman et al.	Hydrochlorothiazide	Hypertension	4 weeks	It did not reduce CRP levels compared to baseline.
Ridker et al. (Val-MARC)	Hydrochlorothiazide + Valsartan × Valsartan	Hypertension	6 weeks	Hydrochlorothiazide + valsartan did not reduce CRP levels compared to treatment alone with valsartan.
Eriksson et al.	Hydrochlorothiazide × candesartan × placebo	Hypertension	12 weeks	Diuretics were associated with higher CRP levels compared to treatment with candesartan or placebo.

CRP: C-reactive protein.

**Table 5:** Effect of different classes of antihypertensive drugs on endothelial function and inflammation.

Drug Classes Antihypertensive	Effect on Endothelial Function and Inflammation
ARB	They have been shown to improve endothelial dysfunction and reduce levels of inflammatory markers.
ACEI	They have shown beneficial effects on endothelial function, but there are few studies evaluating the effect of this class on inflammatory markers.
CCB	They have shown beneficial effects on endothelial function, but there are few studies evaluating the effect of this class on inflammatory markers.
β-blockers	β-blockers of first and second generation did not demonstrate beneficial effects on endothelial dysfunction and inflammation. β-blockers, have shown benefits in endothelial function, however, there are few studies that have evaluated the effect of these drugs on the levels of inflammatory markers.
Diuretics	No studies were found that evaluated the effect of diuretics on endothelial function. They showed no benefit in reducing inflammatory markers.
